# Spatially and spectrally engineered spin-orbit interaction for achromatic virtual shaping

**DOI:** 10.1038/srep09822

**Published:** 2015-05-11

**Authors:** Mingbo Pu, Zeyu Zhao, Yanqin Wang, Xiong Li, Xiaoliang Ma, Chenggang Hu, Changtao Wang, Cheng Huang, Xiangang Luo

**Affiliations:** 1 State Key Laboratory of Optical Technologies on Nano-Fabrication and Micro-Engineering, Institute of Optics and Electronics, Chinese Academy of Science, Chengdu 610209, China

## Abstract

The geometries of objects are deterministic in electromagnetic phenomena in all aspects of our world, ranging from imaging with spherical eyes to stealth aircraft with bizarre shapes. Nevertheless, shaping the physical geometry is often undesired owing to other physical constraints such as aero- and hydro-dynamics in the stealth technology. Here we demonstrate that it is possible to change the traditional law of reflection as well as the electromagnetic characters without altering the physical shape, by utilizing the achromatic phase shift stemming from spin-orbit interaction in ultrathin space-variant and spectrally engineered metasurfaces. The proposal is validated by full-wave simulations and experimental characterization in optical wavelengths ranging from 600 nm to 2800 nm and microwave frequencies in 8-16 GHz, with echo reflectance less than 10% in the whole range. The virtual shaping as well as the revised law of reflection may serve as a versatile tool in many realms, including broadband and conformal camouflage and Kinoform holography, to name just a few.

Space-variant distribution of refractive index is the key of light shaping and forms the basis of various realms including optical imaging^1^, subwavelength focusing^2^ and holography^3^,^4^. In the last decade, a vast range of novel devices have been demonstrated with the help of the newly emerging metamaterials^5^,^6^ and transformation optics^7^. Among these diverse applications, the electromagnetic cloaks have attracted special attentions from both the microwave^8^ and optical regimes^9^,^10^,^11^. Nonetheless, only narrow working bandwidth could be achieved at specific polarization state owing to the fundamental bandwidth-delay restriction in the cloaks^12^. In a general sense, the cloaks change the shape seen by electromagnetic detectors thus they actually belong to the context of virtual shaping technology used in the design of stealth aircrafts and ships^13^.

Besides the gradient-index methodology, another promising approach utilizes the phase discontinuity at metasurface to control the motion of light^14^,^15^,^16^,^17^. Owing to the ultra-small thickness, these metasurfaces possess unprecedented advantages over traditional bulk optical elements. In principle, the abrupt change of phase is accompanied with polarization conversions from linear^14^ or circular polarizations^16^ to their cross-polarization states. Due to the equivalency of circular polarization and spin, the latter process is typically referred to as photonic spin-orbit interaction (PSOI)^18^. Nevertheless, previous structures for PSOI are often characterized by low efficiency and/or small working bandwidth.

In this paper, we present for the first time the concept of broadband virtual shaping based on the phase shift induced by PSOI in spatially inhomogeneous and spectrally dispersive metasurfaces. Owing to the abrupt phase change, traditional law of reflection should be revised accordingly. Theoretical and experimental analyses in the optical and microwave frequencies demonstrate unambiguously the versatility of our approach. The results provided here may also stimulate the development of flat optics and electromagnetics^19^.

## Results

### Principle and numerical simulation

**Figure 1:**
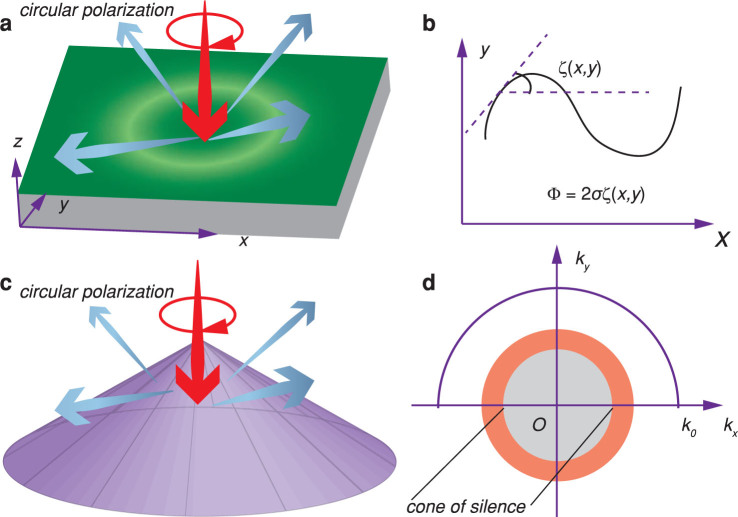
Principle of the virtual shaping based on spin-orbit conversion in space-variant half-wave plates. (a), Schematic of the scattering from a planar surface with space-variant phase distribution under circularly polarized illumination. (b), The phase shift Φ for a rotated half-wave plate under illumination of a circularly polarized wave. (c), The scattered waves from an axicon under normal incidence. (d), Scattering of axicon at normal incidence in the *k*-space.

The modification of electromagnetic shape relies on the phase modulation of scattered wave upon structured surfaces. According to the generalized Snell’s law^14^,^20^, a properly designed reflection phase would force the reflected beam to propagate in well-defined ways with respect to the specular reflection direction (Fig. 1a)^21^. In this way, traditional law of reflection is also broken. In the following, we focus on the geometric phase, a universal but not well-known property resulting from the PSOI in space-variant anisotropic material^22^. As illustrated in Fig. 1b, the reflection phase from a reflective half-wave plate (i.e., no transmission) under circularly polarized illumination can be simply written as Φ(*x*, *y*) = 2*σζ*(*x*, *y*), where *σ* = ±1 denotes the left and right handed circular polarizations (LCP and RCP) and *ζ* defines the orientation angle of the half-wave plate. It is clear that the geometric phase is independent on the working wavelength, thus broadband performance can be expected if an achromatic half-wave plate is available.

The concept of radar cross section (RCS) was adopted to characterize the performance of virtual shaping^23^. The phase profile needed for RCS reduction can be designed by using iterative Fourier transform for both LCP and RCP input waves^21^. In the simplest case, hereafter a linear planar phase distribution Φ(*r*) = *σk_r_r* was assumed in our design, where *r* and *k_r_* are the radius and the wavevector along it. In this circumstance, the virtual shape becomes an axicon (Figure 1c), with scattered wavevectors spreading like a ring in *k*-space, leaving a ‘cone of silence’ at the center (Fig. 1d).

**Figure 2:**
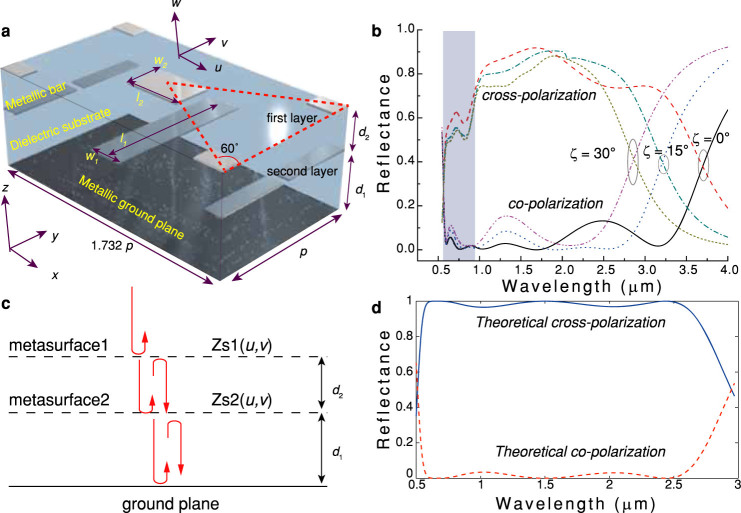
Simulation of the achromatic half-wave plate. (a), Schematic of the unit cells used in simulations. The inclination angle between *u*- and *x*-axis is *ζ*. (b), Calculated reflectances for cross-polarization and co-polarization for different orientation angles under circular polarized illumination. (c), Schematic of the transmission line model for achromatic polarization conversion. (d), Theoretically calculated reflectance for *ζ* = 15°. The effective circuit parameters for the two metasurfaces are *C_u_*_1_ = 3 × 10^−19^ F, *C_v_*_1_ = 5 × 10^−19^ F, *L_u_*_2_ = 1 × 10^−15^ H, *C_u_*_2_ = 1 × 10^−17^ F, *L_v_*_2_ = 3.8 × 10^−13^ H, *C_v_*_2 _ = 1 × 10^−17^ F.

The unit cell of our reflective half-wave plate, composed of two cascaded metasurfaces separated by silica dielectric spacer, is illustrated in Fig. 2a. The metallic bars (Aluminum) in the two cascaded layers are arranged in a hexagonal lattice with C6 symmetry to enhance the polarization adaptability. The achromatic property was obtained by engineering the spectral dispersion of the metasurfaces, with effective inductances and capacitances of^24^,^25^: 
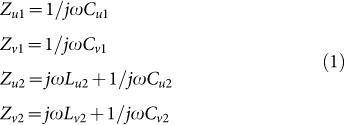
where *L* and *C* stand for the inductances and capacitances, *u* and *v* represent the orthogonal main axes of the half-wave plate, 1 and 2 denote the layer numbers.

It should be noted that the dispersion property is also widely used in other metasurface-based devices, such as filters^26^ and perfect absorbers^27^,^28^,^29^,^30^. Since the principle of these absorbers and polarizers are analogous, the thickness-to-bandwidth ratios of the half-wave plates are expected to have a limit value, similar to that of absorbers^31^: as early as 1906, Planck had revealed that the thickness of electromagnetic absorber must be larger than a limit^32^, which was further clarified by Rozanov in terms of casualty and Kramers-Kronig relation^31^. Most recently, the short wavelength and high dispersive properties of wave at metasurface were utilized to break this limit^29^: It was shown that a 0.3 nm thick tungsten film can absorb almost all the microwave and even terahertz energy under coherent condition. In a similar way, we have shown that the polarization can also be achromatically manipulated using ultrathin metasurfaces^33^.

In our simulations, the reflectance for each unit cell was calculated using CST Microwave Studio with periodic boundary condition. For circularly polarized incidence, the cross-polarized and co-polarized components are illustrated in Fig. 2b. The corresponding geometric parameters were optimized as *p* = 320 nm, *l*_1_ = 295 nm, *l*_2_ = 80 nm, *w*_1_ = 70 nm, *w*_2_ = 50 nm, *d*_1 _ = 120 nm, *d*_2_ = 120 nm. For different orientation angles, all geometric parameters were maintained as that optimized for *ζ* = 0°, leading to a small degradation in the conversion efficiencies. Nonetheless, the mean values of the co-polarized reflectance are less than 0.1 in the wavelengths ranging from *λ* = 600 nm to *λ* = 2800 nm, covering the visible and near infrared spectrum. The decrease in cross-polarized reflectance at wavelengths around 750 nm is owing to the enhanced absorption corresponding to the inter-band transition in Aluminum^34^. As shown in Fig. 2c,d, the transmission line model was used to interpret the results, showing good agreement with the numerical results.

**Figure 3:**
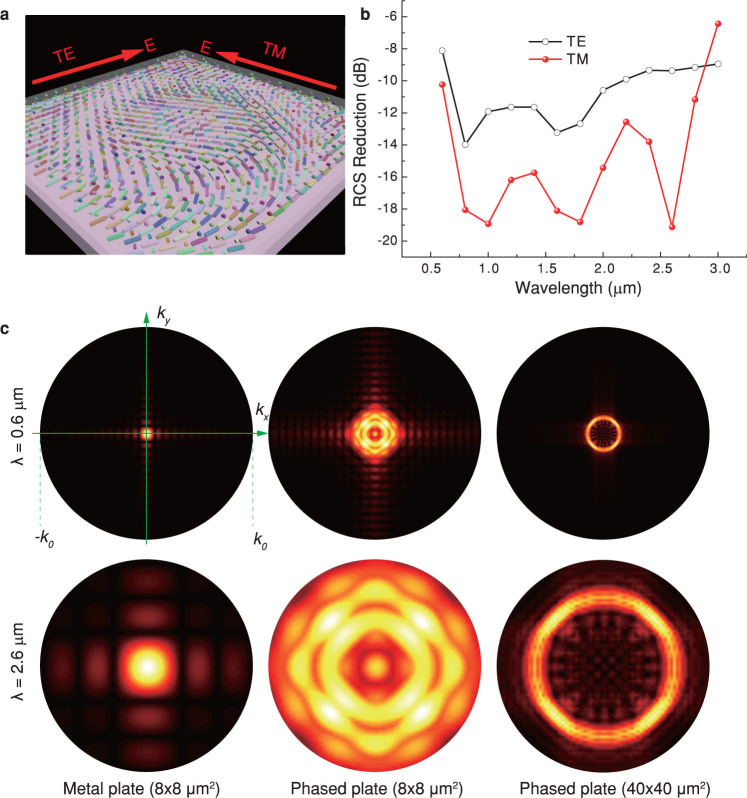
Simulations of the achromatic optical virtual shaping. (a), Schematic of the three dimensional model. The metallic bars are shown with different colors. The incident electric fields for the TE and TM polarizations are depicted. (b), Calculated RCS of a sample with apertures of 8 μm × 8 μm. (c), Calculated diffraction patterns in the *k*-space for a planar metallic plate (the first column), a phased plate with dimensions of 8 μm × 8 μm (the second column), and a phased plate with dimensions of 40 μm × 40 μm (the third column).

Subsequently, the full model, with *k*_r_ = 1.57×10^6^ rad/m and dimensions of 8 μm × 8 μm (Fig. 3a), was numerically simulated, assuming a linear-polarized wave illuminating on the structure at normal incidence. As we expected, the monostatic RCS in 0.6 – 2.8 μm is reduced larger than 10 dB compared with a planar metallic plate for both the transverse electric (TE) and transverse magnetic (TM) polarizations (Fig. 3b). Note that the RCS reduction can be further enhanced by optimizing of the unit cell and phase distributions.

The polarization-independent RCS reduction properties can be understood via analysis for the two individual circular polarization states. For a linear polarized incidence at normal incidence, the reflecting wave can be written in the form of Jones matrix as: 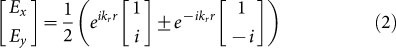
where the sign ± is chosen for *x* and *y* polarized incidences respectively. The first and second items in the right side of equation (2) stand for the contribution of right-handed and left-handed circular polarizations (RCP and LCP). Obviously, the RCS reduction properties are similar for both RCP and LCP, thus our structure can be treated as polarization-independent, in contrast to the intuitive thought about anisotropic material.

In order to get more physical insight into the virtual shaping, the far-field diffraction patterns for a planar metal plate (8 × 8 μm^2^), samples with both small size (8 × 8 μm^2^) and relatively large size (40 × 40 μm^2^) were calculated using vectorial diffraction theory^35^ for *λ* = 600 nm and 2800 nm under the *x*-polarized illumination (Fig. 3c). The diffraction patterns for a larger sample (40 × 40 μm^2^) have a more clear intensity singularity in the center of *k*-space, implying that the device is efficient for objects with either small or large size.

### Experimental demonstration in the microwave regime

**Figure 4:**
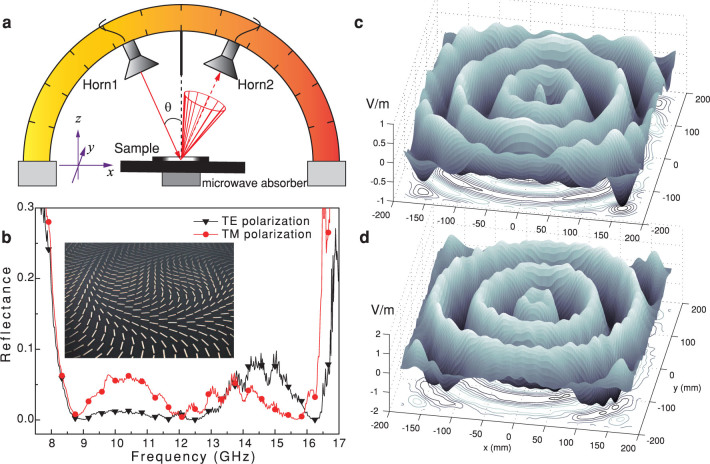
Microwave demonstration of the virtual shaping. (a), Schematic of the measurement setup. (b), Measured results for both TE and TM polarizations. The inset shows the photography of the fabricated sample. (c,d), Scattered electric fields for TM wave at the plane 30 mm above the metasurface for (c) *f* = 8.5 GHz and (d) 16 GHz.

Owing to the scalability of Maxwell’s Equations, the fabrication and characterization of our structure at arbitrary frequencies is feasible. For ease of characterization, the performance was tested for 8–16 GHz in the microwave regime, thus only one layer metasurface is sufficient for the required polarization conversion. The dielectric layer was chosen as FR4 with a permittivity of about 4.4(1 + 0.025*i*) and a thickness of 3 mm. The other geometric parameters were optimized as *p* = 9 mm, *l* = 7.5 mm and *w* = 1 mm. The sample was then fabricated by laser direct writing on a photosensitized FR4 board with a 17 μm copper thickness, and measured in microwave anechoic chamber with a network analyzer (R&S ZVA40). The configuration of the receiving, accepting antennas and the sample is illustrated in Fig. 4a, where the incidence and reflection angles are fixed at 10° to approximate the normal incidence condition. The reflectance was measured for both the TE and TM polarizations, and the corresponding results are shown in Fig. 4b.

To investigate how the electromagnetic wave interacts with the spatially inhomogeneous and spectrally dispersive metasurfaces, the scattered electromagnetic fields at different frequencies were calculated. The incidence plane wave was assumed to be polarized along the *x*-direction (TM) and have an electric field amplitude of 1 V/m. The *y*-components of the total scattering fields were evaluated at the plane 30 mm above the metasurface (Figure 4c, d), presenting perfect agreement with theoretical expectations.

**Figure 5:**
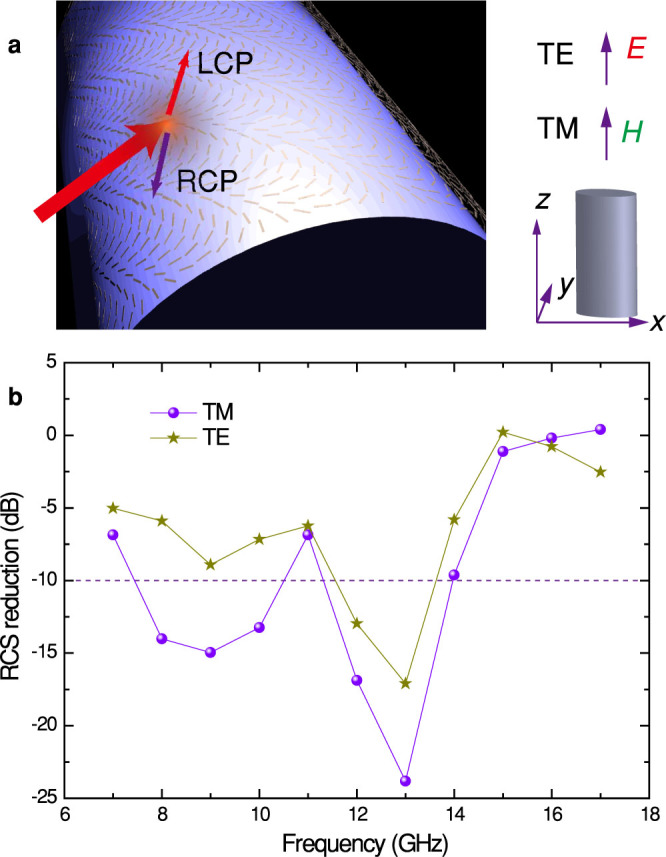
RCS reduction with conformal metasurfaces. (a), Schematic of the spin-orbit conversion in the curved metasurface on a metallic cylinder. The insets show the definitions of TE and TM polarizations. (b), Calculated broadband RCS reduction for TE and TM polarizations at normal incidence along *y*-direction.

Finally, we would like to extend the concept of virtual shaping to non-planar surfaces^36^. For example, the RCS of a cylinder can be dramatically reduced by covering our metasurfaces on it (Fig. 5a). The metallic cylinder in our design has a radius of *R* = 90 mm and height of *h* = 360 mm. The parameters for the metasurface unit cell are identical to the above design. The geometric phase distribution on the metasurface was designed to be Φ = *k_z_z* + *k*_φ_φ, where *k_z_* = *k_φ_* = 104.7 rad/m. As shown in Fig. 5b, the RCS reductions for TE and TM polarizations were calculated under normal incidence along the *y*-direction. We note that the reduction amount is a bit smaller than the planar case, which is possibly stemming from the non-optimized phase profile and the fact that the RCS of a cylinder itself is smaller than its planar counterpart. Nevertheless, the conformal metasurfaces provide a new sight into the virtual shaping of non-planar objects.

## Discussions

In summary, we proposed and demonstrated the concept of broadband virtual shaping at the visible, infrared and microwave spectrum by tailoring the spatio-temporal property of spin-orbit interaction in cascaded metasurfaces with either planar or curved topography. This scheme decouples the problem of broadband virtual shaping into the design of achromatic half-wave plate and iterative Fourier transformation design. Resorting to the dispersion engineering techniques in metamaterial-based polarizers, the bandwidth is dramatically enhanced, despite the thickness to bandwidth ratio may be analogous to that of broadband absorber^31^. Although only Bessel-type phase distribution is analyzed here, in principle, the performance of virtual shaping can be further improved by more elaborate design of the phase distribution carried by the metasurfaces. The design principle provides a new route for the control of electromagnetic wave for applications ranging from laser beam shaping to 3D holographic display and conformal camouflage^37^.

## Methods

### Numerical simulations

Both the unit cell and the full model were simulated by using commercial software CST Microwave Studios, with unit cell and open boundary conditions, respectively. The diffraction patterns for the theoretical phase distributions were calculated using vectorial diffraction theory^35^, where two-dimensional Fourier transform was applied to obtain the vectorial angular spectra.

### Sample fabrication and characterization

The sample in microwave band was fabricated by using laser direct writing in print circuit board (PCB) technology. The performance of virtual shaping was characterized by the reflection spectra at the specular reflection direction.

## Author Contributions

M.B.P., Z.Y.Z., and Y.Q.W. contributed equally in the design and physical explanation of the experiment. X.L., X.L.M., and C.G.H. prepared the sample. C.T.W. and C.H. characterized the sample in the microwave spectrum. M.B.P. and X.G.L. wrote the manuscript. All the authors contributed to the discussion of the results. X.G.L. proposed the original idea and supervised the project.
